# Ovarian Sac

**Published:** 1829-04

**Authors:** 


### HOSPITAL REPORTS,

*(Principally condensed from various Periodical Publications.)*

---

### OVARIAN SAC.

*Description of an Ovarian Sac, in which Hair was found adhering to the Integuments of an extremely small Osseous Body, of an orbicular form, not unlike the Skull externally, and endowed with Organic Life.* By A. Reynaud. (La Charité.)

Although it is not very unusual to find collections of hair accidentally developed in different parts of the body, and more particularly in the ovaries, the number of observations of this kind is not so considerable but that science may still profit by the discovery of a new fact; and it is with this conviction that we detail the following curious case.

A woman, twenty-four years old, was admitted into the hospital with severe ulceration of the breasts, in which large abscesses had formed, in consequence of very high inflammation which supervened early after delivery. She eventually died of this complaint, worn out by the excessive suppuration, pain, and fever with which it was attended. She had, within the twenty-seven months that preceded her illness, twice conceived, and brought forth a live and well-formed child each time.

The body was examined twenty-four hours after death, when the following anomalies were recorded:

The left ovary was found as large as an orange, free from unnatural adhesions, and situated in the pelvis anterior to the uterus, for which it was at first mistaken. It was thought surprising that the uterus should be so unusually large in the unimpregnated state, so long after delivery; but, on further investigation, it was suggested that this mass, since it externally resembled the uterus, might possibly be a case of extra-uterine pregnancy. Accordingly, the examination of the contents of this tumor was conducted with the greatest care and circumspection; and, on dividing it longitudinally, in a direction opposite to that in which the peritoneal covering of the ovary is inserted, a cavity was laid open, almost completely filled by a large wad of hair. These hairs were entangled together in the most complicated manner imaginable, and the space between them was filled with a substance like fat, semifluid, of a white gray, strongly scented, and somewhat resembling the sebaceous matter which, in some diseases of the hairy scalp, is secreted by the follicles of that part. This matter had been deposited on the internal surface of the cyst, where it was thicker than in other parts, and had the appearance of a false membrane. The extremities of many of the hairs were inserted into this membrane, and, on a first glance, they appeared to derive their origin thence; but, on detaching them from it, their extremities were not found to be bulbous, but, on the contrary, very small, and much less like roots than points of hair: besides, some of these hairs passed only through this membranous substance, while both their ends were traced into some other part of the tumor.

These hairs were not all either of the same length or colour: a great number of them were flaxen, or indeed nearly red; they were generally short and slender; they were easily separated, without breaking, or, by their resistance, evincing in any way that they adhered to any part; whilst others, on the contrary, formed dark brown masses, from which, when carefully unravelled, some hairs might be drawn out more than a foot long: these were bigger and more hard than the first. Further investigations were made, to discover, if possible, in some point of this anomalous production, either the remnants of a fœtus, or some other portion of organized matter from which its origin might be deduced.

From a certain point of the internal surface of the ovary, a kind of fibrous pedicle was seen coming off, and penetrating deeply into the mass of hair; and, by careful dissection, it was traced into a substance of an irregular form, but nearly round, of the size of a walnut, bony towards its centre, enveloped in a membrane, and terminating in a point, whence two firm, fibro-cellular prolongations were sent off, to be separately inserted into a part of the sac diametrically opposite to that from which the pedicle arose. The membrane which covered it for the most part resembled the scalp in children affected with porrigo, after that the crusts have been detached by the use of detergent lotions; its external surface was moist, reddish, felt greasy, and gave insertion to a considerable number of hairs: when pulled out, a bulbous root was found attached to each, and amongst these hairs were the longest and darkest of all those which were contained in the ovary. Other hairs, of a lighter colour and much shorter, also adhered to its surface, which, in the intervals between the different hairs, presented a number of small indentations. Underneath this sort of skin there was a greasy membrane, extremely thin, but distinct, and very similar to that which lines the internal surface of the skin of the cranium. The bulbs of the hair were lodged in the substance of this second membrane, which closely adhered to this almost formless bony mass above described, and which could not certainly, by any effort of the imagination, be fancied to possess the smallest resemblance to the human fœtus. This unnatural production was convex where the hairs were found adhering, and in the opposite direction rather concave; in which part its investing membrane was of a serous kind, and did not contain a single hair. A blowpipe being insinuated below it by means of a small incision, it was dilated by the air thus introduced, and assumed the form of a bag, terminating in a cul-de-sac about the middle of the fibrous cords or prolongations already mentioned. Two or three blood-vessels were very distinctly seen extending along one of these cords towards the osseous mass, and a great many very minute ramifications of them were distributed over the internal surface of that portion of the integument to which the hairs were found attached.

The pouch in which all these extraordinary objects were discovered consisted of three distinct coats. The external one was nothing but the fibro-serous covering of the ovary itself: it was internally lined by cellular membrane, vascular and more thick; which, lastly, was in contact with a thin, smooth membrane.

The right ovary exhibited nothing remarkable, excepting a little unevenness of its external surface, which was owing to several small cicatrices. The fallopian tubes were pervious; the uterus had resumed its natural dimensions, and its interior was red and vascular. Divided internally, its substance was found rather soft, and of a yellowish colour.

A greater or smaller quantity of hair, either by itself or together with teeth and other bony productions, have been repeatedly found in various parts of the body, and has been the subject of many learned discussions. Meckel* has written an excellent dissertation on this subject, in which he has given an account of the different opinions which have been entertained by physiologists at various periods concerning the formation of these substances, with a critical estimate of their respective merits.

